# Management of a Gastrobronchial Fistula Connected to the Skin in a Giant Extragastric Stromal Tumor

**DOI:** 10.1155/2015/204729

**Published:** 2015-07-28

**Authors:** Emilio Muñoz, Fernando Pardo-Aranda, Noelia Puértolas, Itziar Larrañaga, Judith Camps, Enrique Veloso

**Affiliations:** Hospital Universitari Mutua Terrassa, 08221 Barcelona, Spain

## Abstract

*Introduction*. Gastrointestinal stromal tumors first treatment should be surgical resection, but when metastases are diagnosed or the tumor is unresectable, imatinib must be the first option. This treatment could induce some serious complications difficult to resolve. *Case Report*. We present a 47-year-old black man with a giant unresectable gastric stromal tumor under imatinib therapy who presented serious complications such as massive gastrointestinal bleeding and a gastrobronchial fistula connected with the skin, successfully treated by surgery and gastroscopy. *Discussion*. Complications due to imatinib therapy can result in life threatening. They represent a challenge for surgeons and digestologists; creative strategies are needed in order to resolve them.

## 1. Introduction

Gastrointestinal stromal tumors (GIST) are originated in the interstitial cell of Cajal [1] and have initial intramural growth. They can be intraluminal or extraluminal reaching a progressive bulky size and becoming giant tumors, especially in the stomach, which represents around 60% of all GIST [2].

In these giant gastric tumors necrotic intratumor cavities communicated to gastric lumen are quite common and can be infected, cause bowel obstruction, suffer a spontaneous rupture, or even bleed [3–6].

Some tumors can be unresectable because of their large size that affects nearby organs or metastatic disease at the time of clinical presentation. Imatinib plays an important role in these situations but may not always give the expected response. Besides acquired resistances [7, 8], it could induce serious complications because of massive necrosis. Although rare, these complications represent a therapeutic challenge for surgeons, like the case we present.

## 2. Case Report

**Figure 1:**
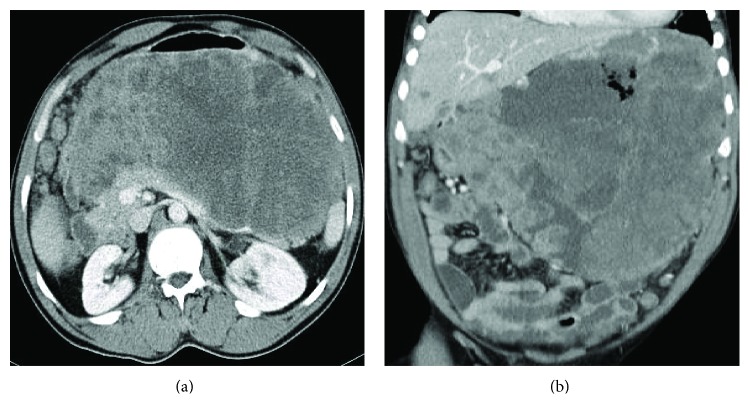
(a) Coronal section: giant GIST with aerial bubbles inside. (b) Axial section: giant gastric GIST.

**Figure 2:**
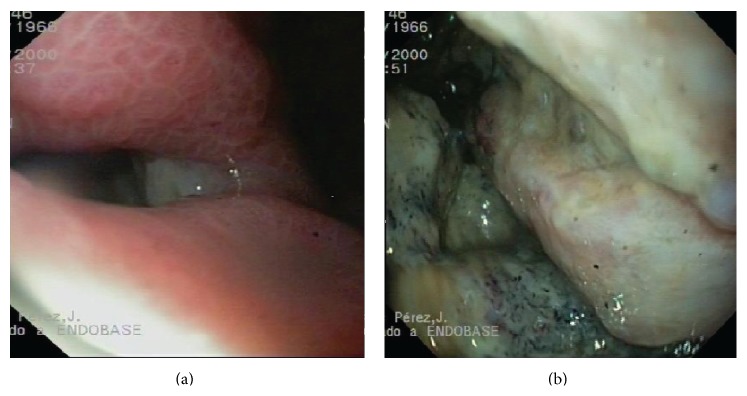
(a) Gastric fistula hole. (b) Necrotic intratumoral cavity.

The patient is a 47-year-old black male, with no medical history reported, diagnosed with a giant, cavitated, and unresectable gastric stromal tumor (GIST) (Figures 1(a) and 1(b)) with high mitotic index and* c-kit gene* exon 11 mutations, fistulized to the stomach lumen (Figures 2(a) and 2(b)), and liver metastases. The patient was started to be treated in March 2013 with imatinib 400 mg daily for a month. In May 2013, he was admitted to the Emergency Department due to melena. At the time of his admission the hemoglobin was 5 g/dL, blood pressure was 80/40 mmHg, and heart rate was 90 lpm.

Once hemodynamically stable, a gastroscopy was performed, with findings of a stomach full of blood clots and heavy bleeding coming from the tumor cavity, which was also full of clotted blood and its irregular walls were oozing blood that was not controllable with argon beam; therefore, an urgent laparotomy was carried out to achieve hemostasis. Intraoperatively, a huge intra-abdominal mass was found blocking nearly three-quarters of the abdominal cavity with strong fibrotic adhesions to the diaphragm, abdominal viscera, and abdominal wall, which made it inadvisable to resect. A gastrotomy was performed through anterior gastric wall to remove all blood clots and the intragastric fistula hole was closed with interrupted absorbable suture. The tumor cavity was opened through the softer tumor area and complete hemostasis was achieved using diathermia and homeostatic biological sheets (Tachosil, Surgicel). A closed drainage was left in the tumor cavity. Patient recovered uneventfully and was discharged on postop day 14, in order to continue his treatment in the outpatient clinics. In August 2013, he started suffering from vomiting and imatinib was reduced to a dose of 300 mg daily, being well tolerated.

The patient was readmitted to the Emergency Department four months later, with fever (37.8°C), weight loss, persistent cough, pus leaking through the old drainage scar, and left pleural effusion. A sample of sputum was obtained. CT imaging showed persistence of liver metastases without relevant changes, but the primary tumor had experienced a very impressive shrinkage. The CT scan also showed a left pleural effusion with atelectasis and a left subphrenic abscess that was not suitable for percutaneous drainage; therefore, a surgical debridement and drainage were indicated.

Intraoperatively, a complete blockage of the supramesocolic abdomen was found. Several biopsies were taken (all of which were negative for GIST). Following the old drain tract, a collection with chronic appearance was debrided and sent for culture. A double closed drainage was left in the abscess cavity and a feeding jejunostomy was placed. Microbiological culture of the sputum and collection samples were positive for MRSA and* Candida*. The patient was treated with appropriate antibiotics and antifungals.

**Figure 3:**
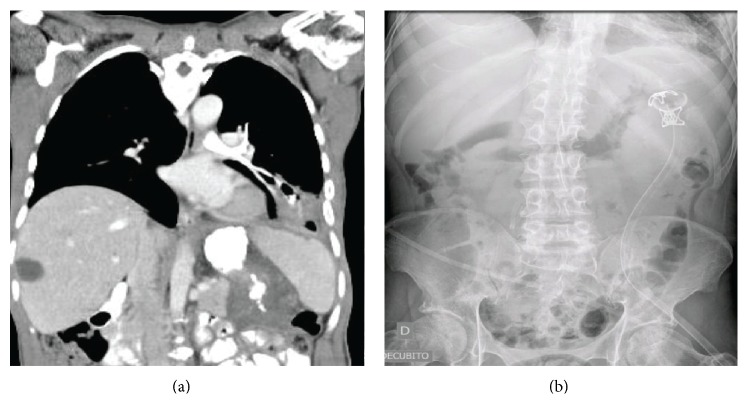
(a) Left bronchial tree full of contrast from stomach and abscess cavity. (b) PEG tube from stomach to the skin cavity isolating bronchial fistula tract.

Postoperatively, after an early clinical improvement, we realized that performing flushes through the double drain tube caused intense cough. A left bronchial fistula (Figure 3(a)) and a gastric fistula were confirmed. Both were connected with the abscess cavity and skin through the abscess drainage. The attempt to close twice a small gastric hole using over-the-scope clips (OTSC) not only was unsuccessful but also made the gastric fistula bigger, worsening the bronchial symptoms.

As the performance status was inappropriate for a left thoracotomy and taking into account the complete frozen upper abdomen, it was decided to isolate the bronchial fistula organizing a controlled gastrocutaneous fistula tract. Using a pediatric gastroscope, a transgastric abscess cavity examination was performed. Once in the abscess cavity, the tip of the drain was identified, and as it was being removed, it was followed by the pediatric endoscope until the skin was reached. A nylon guide was introduced and a 20 Fr percutaneous gastrostomy (PEG) tube was placed from stomach to skin (Figure 3(b)).

**Figure 4:**
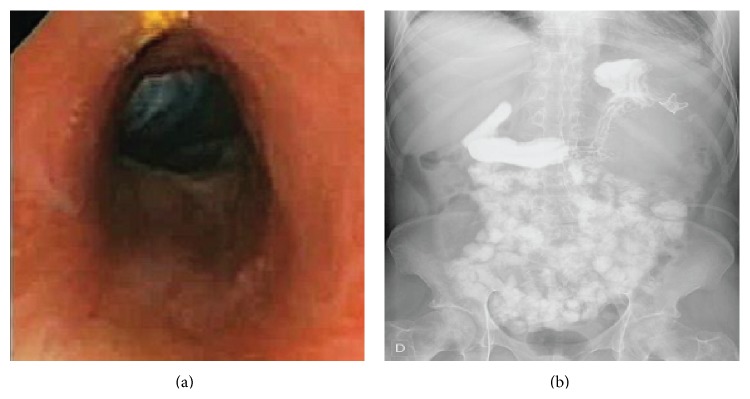
(a) Uniform duct fistula 18 cm long from stomach to the skin. Obtained with pediatric gastroscope. (b) Gastrografin transit without any leakage. OTSC still remains near the old fistulous opening.

Progressive hyperproteic enteral feeding was given up to 5,25 kcal/kg/hour. Two weeks later, the cough, pleural effusion, and atelectasis had disappeared. In 2 months, the patient had gained 12 kg (to reach 54 kg), and a regular 18 cm long gastrocutaneous fistulous tract had been achieved (Figure 4(a)). Gastrografin transit showed no leakage (Figure 4(b)) and once the PEG tube was removed, the fistula tract closed completely in 6 days and the patient was discharged with oral intake and imatinib treatment. Although liver metastases are still in regression, the abdominal mass has not recurred and the patient is having a good quality of life sixteen months later.

## 3. Discussion

Imatinib therapy is justified for locally advanced GIST and for metastatic, recurrent, and unresectable disease [8]. It should be used indefinitely as long as the tumor does not progress and patient tolerance permits [9, 10].

Complications under imatinib therapy due to tumor necrosis appear when the treatment has its higher effect during the first six months of therapy [11, 12].

We found in the literature some unusual cases of GIST bleeding caused by imatinib therapy [13]. Nevertheless, complications such as gastrobronchial fistula are exceptional, and we have not found any similar case in the literature. Probably, necrosis induced by imatinib must play an important role, but we cannot be sure about the exact pathophysiology of this serious complication. Two explanations may be posited: (a) gastric GIST was invading diaphragm and left lung, and necrosis tumor resulted in the formation of the fistula; (b) GIST are rarely infiltrative tumors, so that we believe that communication between the gastric lumen and tumor necrotic area was set up as a result of gastric wall necrosis progression under imatinib therapy. Consequently, an infection in left subphrenic space occurred and turned out into chronic abscess that drained through the weakest area such as diaphragm adhered to the lung.

Regardless of the pathophysiology, the fact was that we have a complex disease like a gastrobronchial fistula, also connected to the skin through a drainage tube, in a patient with poor general condition.

Taking into account patient's performance status and the abdominal blockade, surgical approach was not the best option. Bad as this situation was, we thought that the most feasible solution should be as simple as possible.

“Over-the-scope clip” (OTSC), which has demonstrated its effectiveness in the treatment of similar gastric holes [14, 15], seemed to be an easier way to fix it but failed, maybe due to gastric wall inflammation. Alternatively, we attempted to convert this complex triple fistula to a simple fistula without any intermediate cavity between the stomach and the skin to isolated bronchial communication and achieve a spontaneous closure.

Once we checked that there was no distal bowel obstruction, a pediatric gastroscope was used to see the fistula tract from the stomach to the skin and a PEG tube properly adjusted to the fistula was placed.

Finally, with appropriate feeding support over four months, expected evolution was achieved.

## 4. Conclusion

Complications due to imatinib therapy, such as gastrobronchial fistula, can result in life threatening; surgeons and digestologists should be aware of these uncommon complications.
